# Analysis of Human Dopamine D_3_ Receptor Quaternary Structure*

**DOI:** 10.1074/jbc.M114.630681

**Published:** 2015-04-30

**Authors:** Sara Marsango, Gianluigi Caltabiano, Chantevy Pou, María José Varela Liste, Graeme Milligan

**Affiliations:** From the ‡ Molecular Pharmacology Group, Institute of Molecular, Cell, and Systems Biology, College of Medical, Veterinary and Life Sciences, University of Glasgow, Glasgow G12 8QQ, Scotland, United Kingdom and; § Laboratori de Medicina Computacional, Unitat de Bioestadística, Facultat de Medicina, Universitat Autònoma de Barcelona, 08193 Bellaterra, Spain

**Keywords:** G protein-coupled receptor, dopamine receptor, homodimer, tetramer, GPCR quaternary structure, molecular modeling, fluorescence resonance energy transfer

## Abstract

The dopamine D_3_ receptor is a class A, rhodopsin-like G protein-coupled receptor that can form dimers and/or higher order oligomers. However, the molecular basis for production of these complexes is not well defined. Using combinations of molecular modeling, site-directed mutagenesis, and homogenous time-resolved FRET, the interfaces that allow dopamine D_3_ receptor monomers to interact were defined and used to describe likely quaternary arrangements of the receptor. These were then compared with published crystal structures of dimeric β_1_-adrenoreceptor, μ-opioid, and CXCR4 receptors. The data indicate important contributions of residues from within each of transmembrane domains I, II, IV, V, VI, and VII as well as the intracellular helix VIII in the formation of D_3_-D_3_ receptor interfaces within homo-oligomers and are consistent with the D_3_ receptor adopting a β_1_-adrenoreceptor-like quaternary arrangement. Specifically, results suggest that D_3_ protomers can interact with each other via at least two distinct interfaces: the first one comprising residues from transmembrane domains I and II along with those from helix VIII and a second one involving transmembrane domains IV and V. Moreover, rather than existing only as distinct dimeric species, the results are consistent with the D_3_ receptor also assuming a quaternary structure in which two transmembrane domain I-II-helix VIII dimers interact to form a ”rhombic” tetramer via an interface involving residues from transmembrane domains VI and VII. In addition, the results also provide insights into the potential contribution of molecules of cholesterol to the overall organization and potential stability of the D_3_ receptor and possibly other GPCR quaternary structures.

## Teaser

**Background:** The dopamine D_3_ receptor can form dimers/oligomers, but the molecular basis for this is poorly defined.

**Results:** Molecular modeling, mutagenesis, and analysis of inactive state receptor crystal structures allowed assessment of models of receptor organization.

**Conclusion:** The dopamine D_3_ receptor can assume different dimeric and a rhombic tetrameric arrangements.

**Significance:** These findings provide understanding of the molecular basis of D_3_ receptor quaternary structure.

## Introduction

It is now well accepted that, as well as members of the class C, glutamate receptor family, class A, rhodopsin-like G protein-coupled receptors (GPCRs)^5^ can form dimers and/or higher order oligomers (1–3). Based on this, there is great interest in how such protein-protein interactions might modulate important functions of these GPCRs including maturation, ligand pharmacology, signaling and trafficking (4, 5). The overarching seven transmembrane domain (TMD) architecture of GPCRs and the similarity of the overall domain fold of class A GPCRs observed in various crystal structures suggests potentially conserved interaction interfaces. However, despite numerous molecular, biochemical, and biophysical studies, the molecular basis underlying class A GPCR dimerization and/or oligomerization is unclear and lacks a single unifying hypothesis. Thus, for different family members a range of contact interfaces has been suggested (1–3). Moreover, the capacity to exist as higher order oligomers suggests that multiple contact sites must be present to produce this organization.

Receptors for the neurotransmitter dopamine are GPCRs belonging to the class A family. They are separated into two broad groups based on their principal signaling mechanisms and distribution. The D_1_-like receptors (D_1_ and D_5_) are mainly coupled to stimulatory G proteins and enhance the activity of adenylyl cyclases, whereas D_2_-like receptors (D_2_, D_3_, and D_4_) are largely coupled to inhibitory G proteins and suppress the activity of adenylyl cyclases and modulate a variety of ion channels (6). Dysregulation of dopaminergic neurotransmission in the substantia nigra and in the striatum is implicated in multiple disorders including Parkinson disease, attention deficit hyperactivity disorder, and a group of psychotic disorders including schizophrenia (6). The dopaminergic hypothesis of schizophrenia suggests that this reflects excessive central dopaminergic activity due to changes in dopamine receptors rather than a quantitative change in neuronal dopaminergic activity (7). Conditions such as schizophrenia are treated routinely using ligands with antagonist affinity at the D_2_ receptor, but interestingly, many of these actually have moderate selectivity for the D_3_ receptor over the D_2_ receptor (8–10). Moreover, because of the overlap of ligand recognition between the D_2_ and D_3_ receptors and co-expression of the two receptors in caudate, putamen, and striatum, their individual contributions are challenging to define (11, 12).

Adding complexity to this system is the capability of both D_2_ and D_3_ receptors to form homo- and hetero-oligomers that can also influence dopaminergic neurotransmission (12, 13). Interestingly, in schizophrenia alterations in the proportion of D_2_ receptor monomers *versus* dimers and homomeric D_3_ complexes have been reported (14). Importantly, recent studies indicate that each of these species can co-exist concurrently (15).

Although the capacity of the D_2_ receptor to form homodimers and higher order oligomers has been studied extensively (16–18), less is known about D_3_ receptor homo interactions. The D_3_ receptor is of particular interest as it has been identified as a potential target for drug discovery in the field of drug addiction (19). In the current studies we have employed molecular modeling based on a high resolution, inactive state structure of the human D_3_ (hD_3_) receptor complexed with the antagonist eticlopride (20) to generate different potential models of this receptor in a dimeric arrangement. These models were then assessed after alanine mutagenesis of residues that the models indicated to be potentially involved in dimer interfaces. Homogenous time resolved-FRET (htrFRET) using Tag-Lite^TM^ technology (21) was employed to monitor alterations in the capability of each mutant to form homomers. These studies investigated the roles of regions of TMDs I, II, IV, V, VI, and VII as well as the intracellular helix VIII in the formation of possible interfaces within hD_3_-hD_3_ receptor homo-oligomers. The results obtained are consistent with hD_3_ receptor monomers being able to interact with each other via at least two interfaces of dimerization: the first composed by residues from TMD I and TMD II as well as helix VIII and the second consisting of residues within TMDs IV and V. Furthermore, the data are consistent with the hD_3_ receptor assuming a higher order quaternary structure in which two TMD I-II-helix VIII dimers interact to form a rhombic tetramer via an interface involving residues from TMDs VI and VII. Interestingly, these results also provide insights into the potential contribution of molecules of cholesterol to the overall organization and potential stability of this, and possibly other, GPCRs quaternary structures.

## Materials and Methods

### 

#### 

##### DNA Constructs of the VSV- and SNAP-tagged Human Dopamine D_3_ Receptor (VSV-SNAP-hD_3_)

As described previously, the plasmid pSEMS1–26m (SNAP tag) (22), as supplied by Covalys Biosciences AG (Witterswil, Switzerland), was modified by the addition of a small linker region encoding the metabotropic glutamate receptor 5 signal sequence (MVLLLILSVLLLKEDVRGSAQS) and the VSV epitope tag (YTDIEMNRLGK) between the ClaI and EcoRI sites of the multiple cloning site upstream of the SNAP tag (MCS1). The hD_3_ receptor was PCR-amplified using primers designed to add BamHI and NotI sites to the fragment termini. It was then ligated into the multiple cloning site downstream of SNAP tag of the modified plasmid described above (15).

##### Mutagenesis of VSV-SNAP-hD_3_

The Stratagene QuikChange method (Stratagene, Agilent Technologies, Santa Clara, CA) was used to introduce alterations into VSV-SNAP-hD_3_. Primers utilized for mutagenesis were provided by MWG Operon (Acton, UK). Template DNA was digested with DpnI to leave only the newly synthesized mutated plasmid, and sequencing was carried out to confirm the introduction of the alterations.

##### Cell Culture and Transient Transfection of HEK293T Cells

Human embryonic kidney (HEK) 293T cells were maintained in Dulbecco's modified Eagle's medium supplemented with 0.292 g/liter l-glutamine (Sigma), 1% penicillin/streptomycin mixture (Sigma), and 10% heat-inactivated fetal bovine serum (Gibco, Life Technologies) at 37 °C in a 5% CO_2_ humidified atmosphere. HEK293T cells were transfected using polyethyleneimine (Fluka Analytical, Poole, Dorset, UK). The day before transfection 1 × 10^6^ cells were plated into 60-mm dishes. Plasmid DNA was then combined with polyethyleneimine (in 1:6 ratio) in 250 μl of 150 mm NaCl, thoroughly mixed, and incubated for 10 min at room temperature. Cell medium was changed, and the DNA-polyethyleneimine mixture was added to the medium in a dropwise manner.

##### Cell Lysate Preparation

HEK293T cells transiently transfected with the construct of interest were harvested in ice-cold phosphate-buffered saline (PBS) and lysed in lysis buffer (150 mm NaCl, 0.01 mm Na_2_HPO_4_, 2 mm EDTA, 0.5% *n*-dodecyl-β-d-maltoside (DDM), 5% glycerol, and supplemented with Complete protease inhibitors mixture (Roche Diagnostics)) on a rotating wheel for 30 min at 4 °C. Samples were then centrifuged for 15 min at 21,000 × *g* at 4 °C, aliquoted, and stored at −20 °C until required.

##### Treatment of Cell Lysates

Deglycosylation was performed using peptide-*N*-glycosidase F (Roche Diagnostics) at a final concentration of 0.05 unit/μl for 2 h at 37 °C.

##### Immunoblotting Assays

Cell lysate samples prepared as above were diluted to a final concentration of 2 mg·ml^−1^ in lysis buffer. These were then diluted in Laemmli buffer (5 m urea, 0.17 m SDS, 0.4 m dithiothreitol, 50 mm Tris-HCl, pH 8.0, and 0.01% bromphenol blue) to a final concentration of 1 mg·ml^−1^. Samples were heated at 65 °C for 5 min. 20 μg of protein from each sample was loaded into wells of 4–12% BisTris gels (NuPAGE, Invitrogen) and subjected to SDS-PAGE analysis using NuPAGE® MOPS SDS running buffer (NuPAGE, Invitrogen). After separation, the proteins were electrophoretically transferred onto nitrocellulose membrane, which was then blocked (5% fat-free milk powder in PBS supplemented with 0.1% Tween 20 (PBS-Tween)) for 1 h at room temperature on a rotating shaker. The membrane was then incubated with appropriate primary antibody in 5% fat-free milk powder in PBS-Tween overnight at 4 °C on a rotating shaker. Anti-SNAP antiserum (New England Biolabs Inc., Hitchin, UK) was diluted 1:2000 and anti-α-tubulin antiserum (Sigma) diluted 1:5000. Subsequently, the membrane was washed (3 × 10 min with PBS-Tween) and then incubated for 1 h with the appropriate secondary antibody (horseradish peroxidase-linked donkey anti-rabbit IgG (GE Healthcare) or horseradish peroxidase-linked sheep anti-mouse (GE Healthcare) diluted 1:10,000 in 5% fat-free milk powder in PBS-Tween. After washing (3 × 10 min with PBS-Tween), proteins were detected by enhanced chemiluminescence (Pierce) according to the manufacturer's instructions.

##### Cell Membrane Preparation

HEK293T cells transiently transfected with the construct of interest were harvested in ice-cold PBS, and pellets of cells were frozen at −80 °C for a minimum of 30 min. These were subsequently thawed and resuspended in ice-cold 10 mm Tris, 0.1 mm EDTA, pH 7.4 (TE buffer) supplemented with Complete protease inhibitors mixture (Roche Diagnostics). Cells were homogenized on ice by 40 strokes of a glass on a Teflon homogenizer followed by centrifugation at 200 × *g* for 10 min at 4 °C to remove unbroken cells and nuclei. The supernatant fraction was transferred to ultracentrifuge tubes and subjected to centrifugation at 90,000 × *g* for 45 min at 4 °C. The resulting pellets were resuspended in ice-cold TE buffer and passed through a 25-gauge needle 3 times before being assessed for protein concentration. Membrane preparations were then aliquoted and stored at −80 °C until required.

##### [^3^H]Spiperone Binding Studies on Membrane Preparations

Binding studies were initiated by the addition of 15 μg of cell membrane protein (or 25 μg for poorly expressed mutants) in assay buffer (20 mm HEPES, 6 mm MgCl_2_, 1 mm EDTA, 1 mm EGTA, 40 μm ascorbic acid) to tubes containing [^3^H]spiperone (PerkinElmer Life Sciences) (0.019–14 nm) for saturation binding studies. Nonspecific binding was determined by the addition of 10 μm (+)-butaclamol (Sigma). Reactions were incubated for 2 h at 30 °C and terminated by rapid vacuum filtration though GF/C glass fiber filters (AlphaBiotech, London, UK) followed by 3 washes with ice-cold PBS. The level of radioactivity associated with the filters was quantified using a Tri-Carb 2910 TR scintillation counter (PerkinElmer Life Sciences).

##### htrFRET Studies

Cells transfected with varying amounts of construct cDNA were grown to 100,000 cells per well in solid black 96-well plates (Greiner Bio-One Ltd, Stonehouse, UK) coated with 0.1 mg·ml^−1^ poly-d-lysine (Sigma). The htrFRET assays were conducted using Tag-Lite^TM^ reagents (Cisbio Bioassays, Bagnols-sur-Cèze, France). Briefly, growth medium was replaced with 50 μl of a mixture containing the defined optimal concentrations of Tag-Lite^TM^ SNAP-Lumi4-Tb (10 nm) (as energy donor) and Tag-Lite^TM^ SNAP-Red (100 nm) (as energy acceptor). Plates were incubated for 1 h at 37 °C in a humidified atmosphere (5% CO_2_), and subsequently washed four times in labeling medium (Cisbio Bioassays). Plates with 100 μl/well of fresh labeling medium were then read on a PheraStar FS (BMG Labtech, Ortenberg, Germany) htrFRET compatible reader. Both the emission signal from the Tag-Lite^TM^ SNAP-Lumi4-Tb cryptate (620 nm) and the FRET signal emanating from the acceptor Tag-Lite^TM^ SNAP-Red (665 nm) were recorded after excitation at 337 nm (23).

##### Computational Methods

A modification of the crystal structure of hD_3_ receptor in complex with an antagonist (PDB code 3PBL) (20) was used to build all the dimer and higher oligomer models. As the N-terminal of TMD I in hD_3_ crystals is ∼2 helix turns shorter than all other class A GPCR structures released to date, Modeler 9v8 (24) was used to model a TMD I as long as observed in the turkey β_1_-adrenoreceptor. All released structures featuring cholesterol molecules (β_2_-adrenoreceptor, serotonin 5-HT_2B_ receptor, adenosine A2A receptor, μ-opioid receptor and the P2Y_12_ receptor) were superposed with the hD_3_ model, and the cholesterol(s) was extracted and added to hD_3_ models at the equivalent positions of the structure. Dimers of hD_3_ with an interface including TMD I were constructed based on those observed in the “dimer” crystal structures of the inactive, mutationally stabilized turkey β_1_-adrenoreceptor (PDB code 4GPO) (25). Three different dimers of hD_3_ with an interface including TMD V were based on each of the mouse μ-opioid receptor (26), on human CXCR4 (27), and the turkey β_1_-adrenoreceptor (25). These were built as dimer+dimer, based on overall complementarily of shape, to maximize the buried interface and to avoid contacts between helices and then refined based on experimental data from the mutagenesis studies. That GPCR dimers have room to couple with only a single heterotrimeric G protein implies that a tetramer should be able to interact simultaneously with two functional heterotrimeric G proteins to allow receptor-induced GDP/GTP exchange. The Gα_s_ AH domain of the Gα_s_ subunit within the heterotrimeric G protein (in “empty complex”) undergoes a large rigid-body displacement (28) with respect to its non-coupled GTP-bound form (29), and a similarly large movement has also been reported in the Gα_i_ AH domain of the Gα_i_ subunit (30). Thus, the nucleotide-free G protein requires extra space compared with the GTP-bound conformation. Tetramer models were considered as potentially valid only if they both allowed the simultaneous binding of two heterotrimeric G proteins in their nucleotide-free form, as in the atomic level crystal of the β_2_-adrenoreceptor complexed with nucleotide-free Gα_s_ (PDB code 3SN6) (28) and could account for experimental discrimination (at least simultaneous contribution of the main two “dimeric” interfaces, TMD I-TMD II-helix VIII and TMD IV-TMD V). Modeling figures were generated using PyMOL 1.5.3 (31). The “snake” plot was created using the GPCR-SSFE database (32).

##### Data Analysis

Experiments were performed on at least three independent occasions. All data were quantified and analyzed using GraphPad Prism 5.2. Where appropriate, data are expressed as the mean ± S.E. Statistical analysis was performed by one-way analysis of variance with, where appropriate, the addition of Dunnett's test for multiple comparisons.

## Results

**FIGURE 1.**
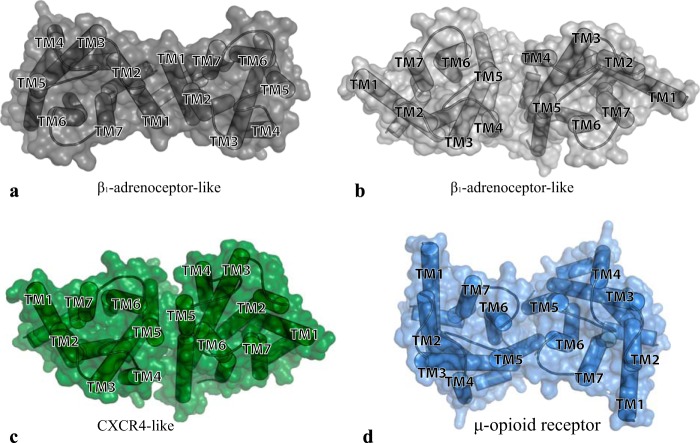
**Molecular models of alternative hD_3_ receptor dimeric arrangements.** hD_3_-hD_3_ interactions may be mediated by interfaces of dimerization composed of residues from TMD I-II and helix VII as observed in a number of different receptors (*a*) and/or TMD IV-V interactions as observed in the turkey β_1_-adrenoreceptor (*b*). TMD V-V interactions, as observed in the CXCR4 chemokine receptor (*c*) or by TMD V-VI interactions (*d*), as observed in the μ-opioid receptor.

In the last few years structures of various class A GPCRs have been released as either asymmetric units of actual dimers (turkey β_1_-adrenoreceptor (25), κ-opioid receptor (33)) or with a computationally estimated biological unit consistent with dimeric organization (CXCR4 chemokine receptor (27), μ-opioid receptor (26), β_2_-adrenoreceptor (34), and P2Y_12_ purinoceptor (35)). Each of these structures shows rather conserved contact interfaces involving interactions between TMDs I, II, and intracellular helix VIII (25, 26, 33). In contrast, less conserved interfaces are observed on the other side of the receptor TMD bundle, with TMD V-TMD VI interactions observed for the μ-opioid receptor (26), TMD IV-TMD V interactions observed in the β_1_-adrenoreceptor (25), and mainly TMD V-TMD V interactions, with contributions from intracellular loop 2, observed in CXCR4 (27). It has been reported that the hD_3_ can form functional dimers/oligomers (15, 36). To explore the molecular basis of this we used the available crystal structure of hD_3_ complexed with the antagonist eticlopride (20) to generate four dimeric models of hD_3_ (Fig. 1). These hypothesized as interfaces (*a*) the broadly conserved TMD I, TMD II, and helix VIII interactions (Fig. 1*a*) observed in many structures, including the β_1_-adrenoreceptor (25), (*b*) the TMD IV-V interface as observed in the β_1_-adrenoreceptor structure (25) (Fig. 1*b*), (*c*) the mainly TMD V-V interface observed in the CXCR4 receptor (27) (Fig. 1*c*), and (*d*) a TMD V-VI interface as observed for the μ-opioid receptor (26) (Fig. 1*d*).

**FIGURE 2.**
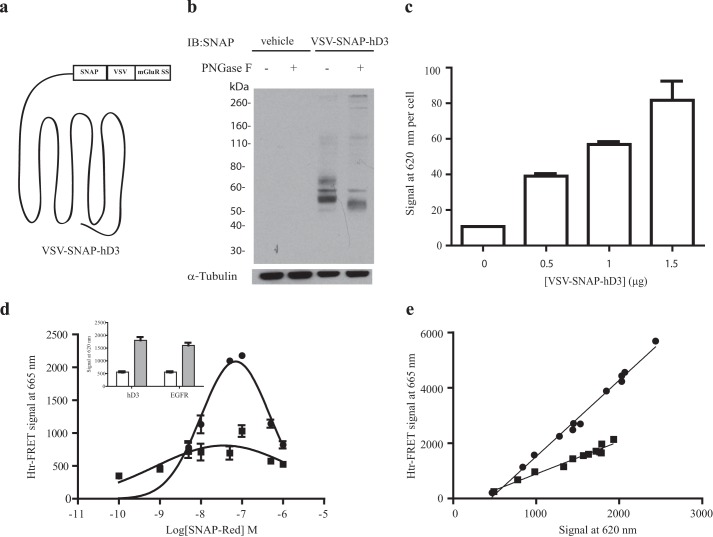
**Organization and expression of a SNAP-tagged form of hD_3_.** *a*, schematic representation of hD_3_ modified at the N terminus by the incorporation of a signal sequence derived from the metabotropic glutamate receptor 5 (*mGluR SS*), the VSV epitope tag, and the SNAP-tag polypeptide to produce the VSV-SNAP-hD_3_ construct. *b*, lysates from HEK293T cells transiently transfected with an empty vector or with VSV-SNAP-hD_3_ were resolved by SDS-PAGE after previous treatment with (+) or without (−) peptide-*N*-glycosidase F (*PNGase F*) and immunoblotted (*IB*) with an anti-SNAP antiserum (*upper panel*) or an anti-α-tubulin antiserum (*lower panel*). *c*, HEK293T cells transfected with increasing amounts of VSV-SNAP-hD_3_ were incubated with the htrFRET energy donor SNAP-Lumi4-Tb (10 nm). SNAP-Lumi4-Tb cell surface binding was determined by fluorescent emission at 620 nm and standardized for cell number. *d*, in cells expressing VSV-SNAP-hD_3_ combinations of SNAP-Lumi4-Tb (10 nm) as energy donors and increasing concentrations of the htrFRET energy acceptor SNAP-Red resulted in a bell-shaped distribution of resonance energy transfer (*circles*) from SNAP-Lumi4-Tb to SNAP-Red. Equivalent experiments were performed on cells expressing VSV-SNAP-EGFR (*squares*) at equal levels of cell surface expression as defined by binding and emission at 620 nm of SNAP-Lumi4-Tb (*d*, *inset*, *open bars* = mock transfection; *filled bars* = corresponding receptor). *e*, htrFRET assays were performed on HEK293T cells transfected with increasing amounts of VSV-SNAP-hD_3_ (*circles*) or VSV-SNAP-EGFR (*squares*) and labeled with an optimal combination of SNAP-Lumi4-Tb (10 nm) and SNAP-Red (100 nm). Cell surface expression (signal at 620 nm) was plotted against energy transfer (signal at 665 nm).

To assess these models a series of htrFRET studies was performed. Initially the hD_3_ receptor was modified at the extracellular N terminus by incorporation of the metabotropic glutamate receptor 5 signal sequence followed by the VSV epitope tag and the SNAP variant of O^6^-alkylguanine-DNA-alkyltransferase. This generated the parental VSV-SNAP-hD_3_ construct (15) (Fig. 2*a*). The SNAP tag sequence allows covalent incorporation of fluorophores into the expressed construct sequence (21). This parental construct was used to transiently transfect HEK293T cells. Lysate from these cells was resolved by SDS-PAGE and immunoblotted with anti-SNAP antiserum (Fig. 2*b*). In such experiments a series of specific immunoreactive species was observed with apparent molecular masses between 65 and 50 kDa. These appeared to represent differentially *N*-glycosylated forms of VSV-SNAP-hD_3_ because pretreatment of the lysate with peptide-*N*-glycosidase F to remove *N*-linked glycans resulted in these species being reduced to a single predominant form that migrated more rapidly in SDS-PAGE (Fig. 2*b*). There was also some evidence of immunoreactive species of substantially lower mobility (Fig. 2*b*).

To assess cell surface delivery of VSV-SNAP-hD_3_, HEK293T cells were transiently transfected with varying amounts of plasmid, and the cells then labeled with the cell impermeant fluorophore SNAP-Lumi4-Tb (10 nm). Subsequent to excitation at 337 nm fluorescence emission at 620 nm, reflecting covalent incorporation of Lumi4-Tb into the extracellular N-terminal domain of VSV-SNAP-hD_3_, demonstrated that increasing levels of cell surface expression of VSV-SNAP-hD_3_ were achieved with increasing plasmid amount over the range assessed (Fig. 2*c*). Fluorescence emission at 620 nm was minimal in empty plasmid-transfected cells (Fig. 2*c*), hence providing excellent signal to background. To examine whether cell surface VSV-SNAP-hD_3_ was present within dimer/oligomer structures, htrFRET was performed using Tag-Lite^TM^ technology. Combinations of SNAP-Lumi4-Tb (10 nm) as the energy donor and varying concentrations of SNAP-Red as the energy acceptor resulted in fluorescence emission at 665 nm, reflecting FRET, after excitation at 337 nm. This is consistent with VSV-SNAP-hD_3_ displaying quaternary organization. As SNAP-Red concentrations were increased, the htrFRET signal initially increased, reached a maximal level, and then subsequently declined (Fig. 2*d*). This is consistent with higher concentrations of SNAP-Red eventually out-competing the available SNAP-Lumi4-Tb for binding to the cell surface population of VSV-SNAP-hD_3_ (Fig. 2*d*). When employing 10 nm SNAP-Lumi4-Tb, maximal htrFRET was obtained with co-addition of 100 nm SNAP-Red (Fig. 2*d*). This combination was then used routinely in subsequent studies. In a parallel set of experiments a modified version of the single TMD epidermal growth factor receptor (EGFR) containing both the VSV and SNAP tags at the extracellular N-terminal region (VSV-SNAP-EGFR) and known to be predominantly monomeric in the absence of agonist activation (37) was employed to define the htrFRET output at 665-nm reported hD_3_-hD_3_ interactions and not simply protein-protein proximity because of the amount of receptor expressed. Transient transfection was optimized to achieve a similar cell surface expression level of this construct, measured by emission at 620 nm after the addition of SNAP-Lumi4-Tb, as for VSV-SNAP-hD_3_ (Fig. 2*d*, *inset*). However, in these cells co-addition of a range of concentrations of SNAP-Red resulted in very little energy transfer (Fig. 2*d*).

After transfection of HEK293T cells with varying amounts of VSV-SNAP-hD_3_ or VSV-SNAP-EGFR, growth in a 96-well microtiter plate, and labeling with the optimized mixture of SNAP-Lumi4-Tb (10 nm) and SNAP-Red (100 nm), fluorescence emission at 620 nm (indicative of cell surface expression of the receptor construct) and 665 nm (reflecting protein-protein interactions) were then measured concurrently and correlated. This produced a linear relationship, indicating constant FRET efficiency over this range of receptor expression (Fig. 2*e*). The slope that characterizes the linear regression generated from VSV-SNAP-hD_3_ (2.78 ± 0.034) or VSV-SNAP-EGFR (1.12 ± 0.049) (mean ± S.E.) was then considered to define the quaternary structure illustrating, respectively, oligomeric and monomeric status of the receptors.

**FIGURE 3.**
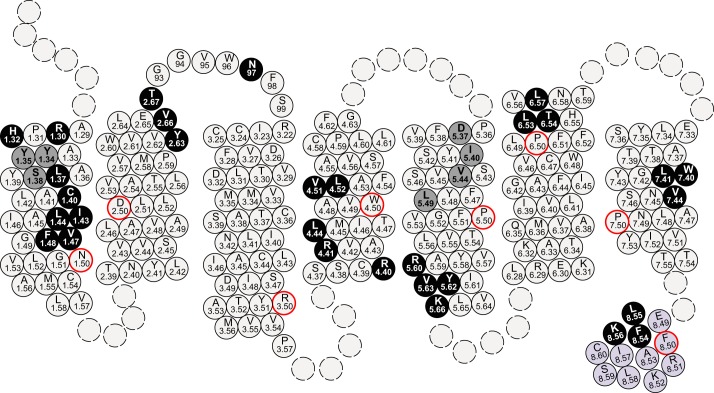
**The topology of VSV-SNAP-hD_3_.** All amino acids of hD_3_ receptor located within TMD I-TMD VII, extracellular loop 1, and helix VIII are shown and designated by the corresponding Ballesteros-Weinstein residue location number. The most highly conserved residue in each TMD (X.50) is shown in a *red circle*. VSV-SNAP-hD_3_ mutants were generated by alanine substitutions. Residues identified to be important for hD_3_-hD_3_ quaternary structure stability as defined in htrFRET studies are shown in *black circles*, whereas residues modified that did not appear to be involved in the formation of a homomeric interface are shown in *gray circles*.

We then used this methodology to consider the models depicted in Fig. 1 with the aim of gaining insights into the most likely organization for the quaternary structure of hD_3_. VSV-SNAP-hD_3_ was used as the template to generate a variety of alanine mutants within TMDs I, II, IV, V, VI, and VII as well as in intracellular helix VIII (Fig. 3). As it was possible that certain of the mutants might result in general unfolding and affect the ligand binding pocket of VSV-SNAP-hD_3_, radioligand binding studies were performed on key mutants. Saturation binding studies were performed on membrane preparations from transiently transfected HEK293T cells using the antagonist [^3^H]spiperone, which has high affinity for the hD_3_ receptor. Apart from the quadruple TMD I mutant, Ile-40,Leu-41,Val-44,Phe-45 VSV-SNAP-hD_3_, [^3^H]spiperone displayed high affinity binding similar to that of the wild type construct (range of values: 0.24–2.47 nm) for each of the mutants subsequently studied (Table 1).

**TABLE 1:** **[^3^H]Spiperone binding affinity of hD_3_ receptor mutants that display altered quaternary structure** The indicated hD_3_ receptor constructs were transiently expressed in HEK293T cells. Saturation [^3^H]spiperone binding studies were performed as described under “Materials and Methods.” Data are presented as the mean **±** S.E. NB, no specific [^3^H]spiperone binding detected over the concentration range employed.

Receptor variant	Ballesteros-Weinstein residue numbering	[^3^H]Spiperone binding *K_D_*
		*nm*
VSV-SNAP-hD_3_		0.55 ± 0.05
Arg-27,His-29,Leu-34,Cys-37	Arg-1.30,His-1.32,Leu-1.37,Cys-1.40	1.23 ± 0.21*^a^*
Ile-40,Leu-41,Val-44,Phe-45	Ile-1.43,Leu-1.44,Val-1.47,Phe-1.48	NB
Tyr-88,Val-91,Thr-92,Asn-97	Tyr-2.63,Val-2.66,Thr-2.67,Asn-97	0.85 ± 0.44
Arg-148,Leu-152,Val-159	Arg-4.40,Leu-4.44,Val-4.51,	2.47 ± 0.40*^a^*
Arg-149,Leu-160	Arg-4.41,Leu-4.52	0.99 ± 0.09*^a^*
Arg-210,Tyr-212	Arg-5.60,Tyr-5.62	1.60 ± 0.14*^a^*
Leu-347,Thr-348,Leu-351	Leu-6.53,Thr-6.54,Leu-6.57	2.03 ± 0.12*^a^*
Trp-370,Leu-371,Val-374	W7.40,Leu-7.41,Val-7.44	0.24 ± 0.15*^a^*
Phe-394	Phe-8.54	1.63 ± 0.18*^a^*
Phe-394,Leu-395,Lys-396	Phe-8.54,Leu-8.55,Lys-8.56	0.68 ± 0.27

*^a^* Statistically different from VSV-SNAP-hD_3_.

**FIGURE 4.**
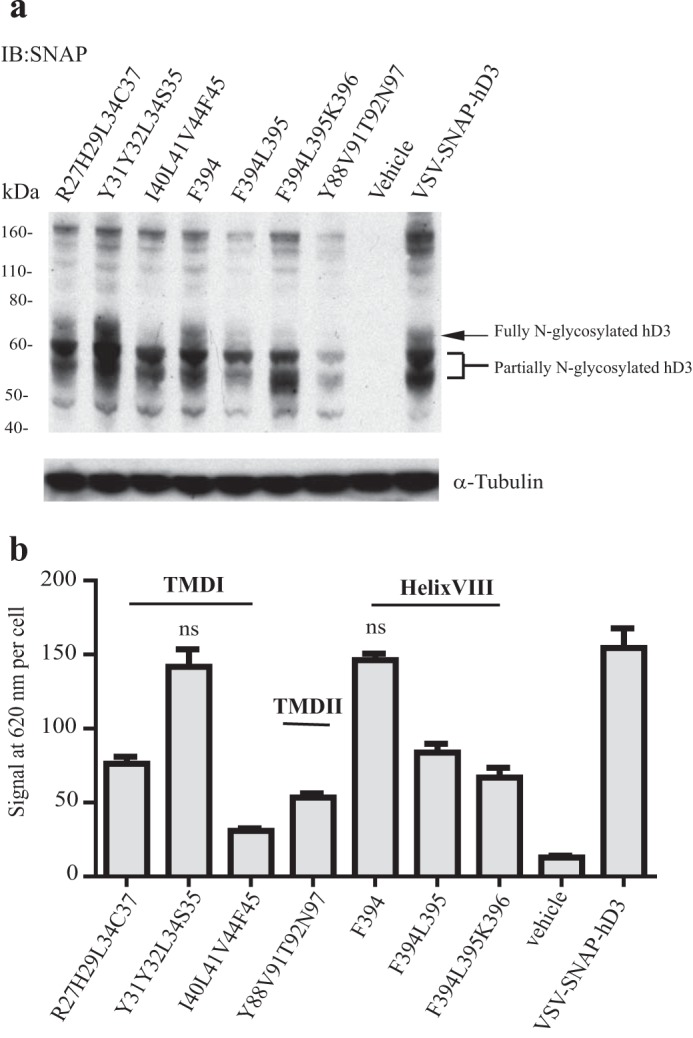
**Expression and cell surface delivery of VSV-SNAP-hD_3_ variants.** *a*, lysates from HEK293T cells transiently transfected with an empty vector, with VSV-SNAP-hD_3_ construct, or each VSV-SNAP-hD_3_ mutant variant of interest were resolved by SDS-PAGE and immunoblotted (*IB*) with anti-SNAP antiserum (*upper panel*) or anti-α-tubulin antiserum (*lower panel*). *b*, HEK293T cells transfected to express wild type VSV-SNAP-hD_3_ or each VSV-SNAP-hD_3_ mutant of interest were incubated with 10 nm SNAP-Lumi4-Tb; cell surface binding was determined as described in Fig. 2.

To assess the model depicted in Fig. 1*a*, three quadruple mutants in TMD I, Arg-27,His-29,Leu-34,Cys-37 (residue positions 1.30, 1.32, 1.37, and 1.40 in the Ballesteros and Weinstein numbering system (38)) VSV-SNAP-hD_3_, Tyr-31,Tyr-32,Leu-34,Ser-35 (1.34, 1.35, 1.37, 1.38) VSV-SNAP-hD_3_, and Ile-40,Leu-41,Val-44,Phe-45 (1.43, 1.44, 1.47, 1.48) VSV-SNAP-hD_3_ were generated. In addition, one quadruple mutant in TMD II, Tyr-88,Val-91,Thr-92,Asn-97 (2.63, 2.66, 2.67, residue 97) VSV-SNAP-hD_3_, and each of a single, double, and a triple mutant in helix VIII, Phe-394 (8.54) VSV-SNAP-hD_3_, Phe-394,Leu-395 (8.54, 8.55) VSV-SNAP-hD_3_, and Phe-394,Leu-395,Lys-396 (8.54, 8.55, 8.56) VSV-SNAP-hD_3_ (Fig. 3) were also generated and studied. Lysates of HEK293T cells transfected with each of these constructs were resolved by SDS-PAGE and analyzed by immunoblotting with an anti-SNAP antiserum. Tyr-31,Tyr-32,Leu-34,Ser-35 VSV-SNAP-hD_3_ and Phe-394 VSV-SNAP-hD_3_ produced a similar pattern of immunoreactive bands and total expression as wild type VSV-SNAP-hD_3_. By contrast, a reduction in the total expression level of Arg-27,His-29,Leu-34,Cys-37 VSV-SNAP-hD_3_ and in the mature, fully *N*-glycosylated form of the mutants Ile-40,Leu-41,Val-44,Phe-45 VSV-SNAP-hD_3_, Tyr-88,Val-91,Thr-92,Asn-97 VSV-SNAP-hD_3_, Phe-394,Leu-395 VSV-SNAP-hD_3_, and Phe-394,Leu-395,Lys-396 VSV-SNAP-hD_3_ was noted (Fig. 4*a*). Cell surface expression of each of these mutants was assessed by the binding of SNAP-Lumi4-Tb (10 nm) and normalized for cell number (Fig. 4*b*). As with many mutants of GPCRs, most of these variants displayed reduced cell surface expression. In particular, a marked reduction of both Ile-40,Leu-41,Val-44,Phe-45 VSV-SNAP-hD_3_ and Tyr-88,Val-91,Thr-92,Asn-97 VSV-SNAP-hD_3_ was observed at the cell surface, whereas more modest, but still significant, reduction of each of Arg-27,His-29,Leu-34,Cys-37 VSV-SNAP-hD_3_, Phe-394,Leu-395 VSV-SNAP-hD_3_, and Phe-394,Leu-395,Lys-396 VSV-SNAP-hD_3_ was recorded (Fig. 4*b*).

**FIGURE 5.**
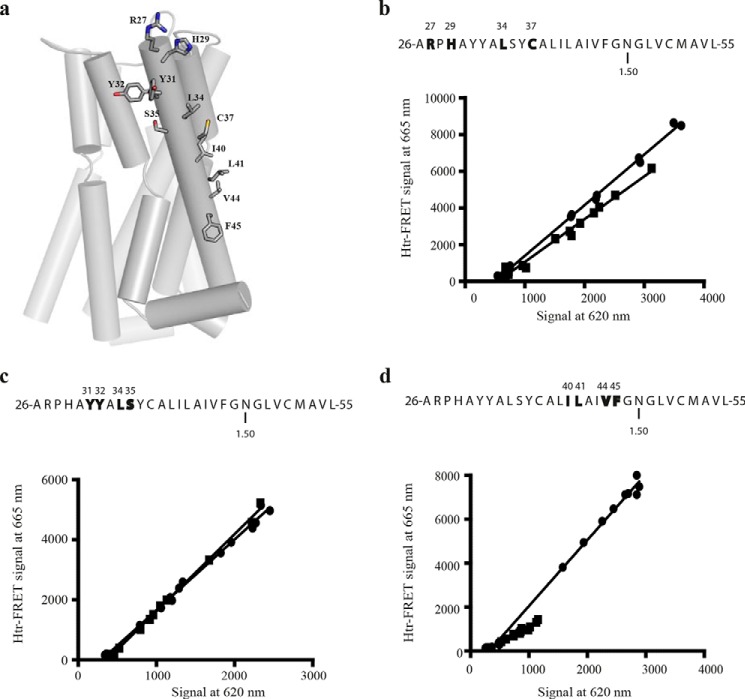
**Role of residues in TMD I in hD_3_-hD_3_ interactions.** *a*, tertiary structure of hD_3_ receptor with TMD I residues that were mutated to alanine shown as sticks. *b–d*, in each case the primary structure of TMD I is presented via the one-letter amino acid code. Amino acids that were replaced with alanine are in *bold* and are denoted by their position in the primary sequence of hD_3_. Asparagine residue 1.50 is also indicated. Representative htrFRET assays performed in HEK293T cells transfected with increasing amounts of Arg-27,His-29,Leu-34,Cys-37 VSV-SNAP-hD_3_ (*b*, *squares*), Tyr-31,Tyr-32,Leu-34,Ser-35 VSV-SNAP-hD_3_ (*c*, *squares*), or Ile-40,Leu-41,Val-44,Phe-45 VSV-SNAP-hD_3_ (*d*, *squares*) were compared with those performed on HEK293T cells transfected with increasing amounts of VSV-SNAP-hD_3_ (*b–d*, *circles*). The plots shown were analyzed by linear regression. See Fig. 10 for analysis of the full data set.

The positions of the residues in TMD I selected for mutagenesis, based on the dimer models, are highlighted within the atomic level structure of the hD_3_ monomer (Fig. 5*A*). Parallel assessment of the ability of these mutants to maintain protein-protein interactions and quaternary structure at the cell surface was conducted via htrFRET assays performed on cells transfected with varying amounts of each mutant and compared directly to the wild type VSV-SNAP-hD_3_ construct. As for VSV-SNAP-hD_3_, each of the TMD I mutants demonstrated both a linear increase in cell surface expression with increasing plasmid amount used to transfect the cells and, over this range, a linear increase of the htrFRET signal at 665 nm (Fig. 5). This indicated that each of the mutants was present within an oligomeric complex. However, the slope of the linear regression of signal at 665 nm/signal at 620 nm was reduced substantially for Arg-27,His-29,Leu-34,Cys-37 VSV-SNAP-hD_3_ (slope = 0.74 ± 0.06-fold of wild type; mean ± S.E.) compared with wild type VSV-SNAP-hD_3_ (Fig. 5*b*). This indicates reduced proximity between the hD_3_ receptor variant protomers and, hence, alteration of receptor oligomer structure. Moreover, although Tyr-31,Tyr-32,Leu-34,Ser-35 VSV-SNAP-hD_3_ did not show an equivalent reduction in the slope of the linear regression (Fig. 5*c*), demonstrating that not all sets of mutations intrinsically interfere with oligomeric organization, for Ile-40,Leu-41,Val-44,Phe-45 VSV-SNAP-hD_3_ this effect was even more marked (0.66 ± 0.02-fold; mean ± S.E.) compared with wild type VSV-SNAP-hD_3_ (Fig. 5*d*) (see later for statistical analysis of the full data set).

**FIGURE 6.**
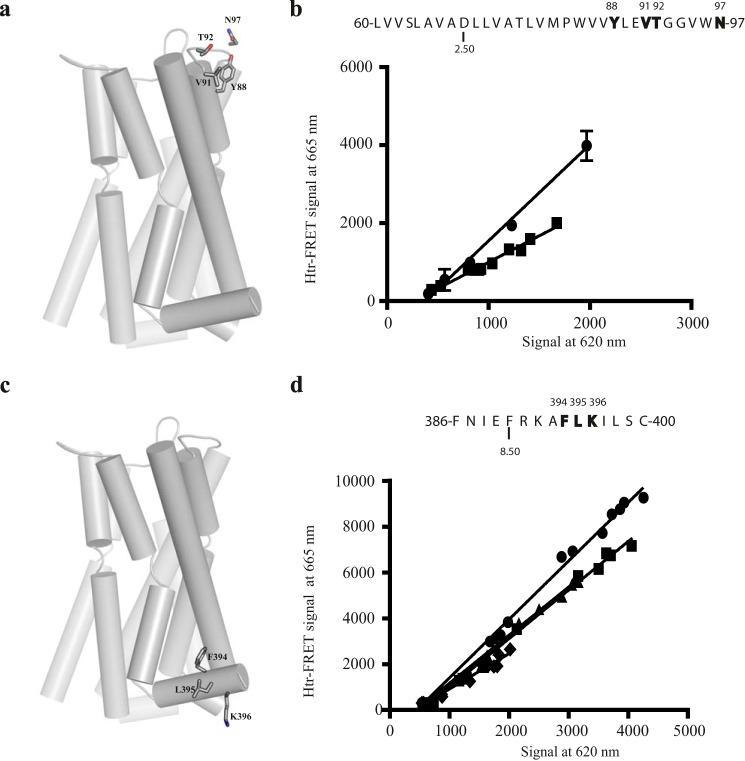
**Role of residues in TMD II and helix VIII in hD_3_-hD_3_ interactions.** *a*, tertiary structure of hD_3_ receptor with TMD II residues that were mutated to alanine shown as sticks. *b*, the primary structure of TMD II is presented via the one-letter amino acid code. Amino acids that were replaced with alanine are in *bold* and are denoted by their position in the primary sequence of hD_3_. Aspartic acid 2.50 is also indicated. HtrFRET assays performed in HEK293T cells transfected with increasing amounts of VSV-SNAP-hD_3_ (*circles*) or Tyr-88,Val-91,Thr-92,Asn-97 VSV-SNAP-hD_3_ (*squares*). *c*, the primary structure of helix VIII is presented via the one-letter amino acid code; amino acids that were replaced with alanine are in *bold. d*, HtrFRET assays performed in HEK293T cells transfected with increasing amounts of VSV-SNAP-hD_3_ (*circles*), Phe-394 VSV-SNAP-hD_3_ (*squares*), Phe-394,Leu-395 VSV-SNAP-hD_3_ (*triangles*), or Phe-394,Leu-395,Lys-396 VSV-SNAP-hD_3_ (*diamonds*). The plots shown were analyzed by linear regression. See Fig. 10 for analysis of the full data set.

In a similar manner mutants in TMD II generated based on modeling of the receptor were mapped on to the receptor structure (Fig. 6*a*). htrFRET assays performed with the TMD II quadruple mutant Tyr-88,Val-91,Thr-92,Asn-97 VSV-SNAP-hD_3_ (Fig. 6*b*) revealed that the slope of the linear regression line was also reduced compared with that for the wild type receptor (0.68 ± 0.07-fold of wild type; mean ± S.E.) (Fig. 6*b*). Finally within this set of experiments, cell surface delivery and htrFRET assays performed with the helix VIII mutants Phe-394 VSV-SNAP-hD_3_, Phe-394,Leu-395 VSV-SNAP-hD_3_, and Phe-394,Leu-395,Lys-396 VSV-SNAP-hD_3_ (Fig. 6*c*) revealed that the slope of the linear regression for each of these was reduced compared with the wild type receptor (0.70 ± 0.04, 0.60 ± 0.12, and 0.49 ± 0.04; mean ± S.E., respectively) (Fig. 6*d*). However, although the reduction in slope recorded for Phe-394,Leu-395 VSV-SNAP-hD_3_ was not significantly different from that observed for Phe-394 VSV-SNAP-hD_3_, the effect on the slope for Phe-394,Leu-395,Lys-396 VSV-SNAP-hD_3_ was significantly greater than for Phe-394 VSV-SNAP-hD_3_ (Fig. 6*d*). This indicates roles of both Phe-394 and Lys-396 in the formation of an interface for hD_3_-hD_3_ interactions. Overall, these results indicated an interface of oligomeric organization that involves residues from both the extracellular and cytoplasmic side of TMD I, the extracellular side of TMD II, and from helix VIII.

**FIGURE 7.**
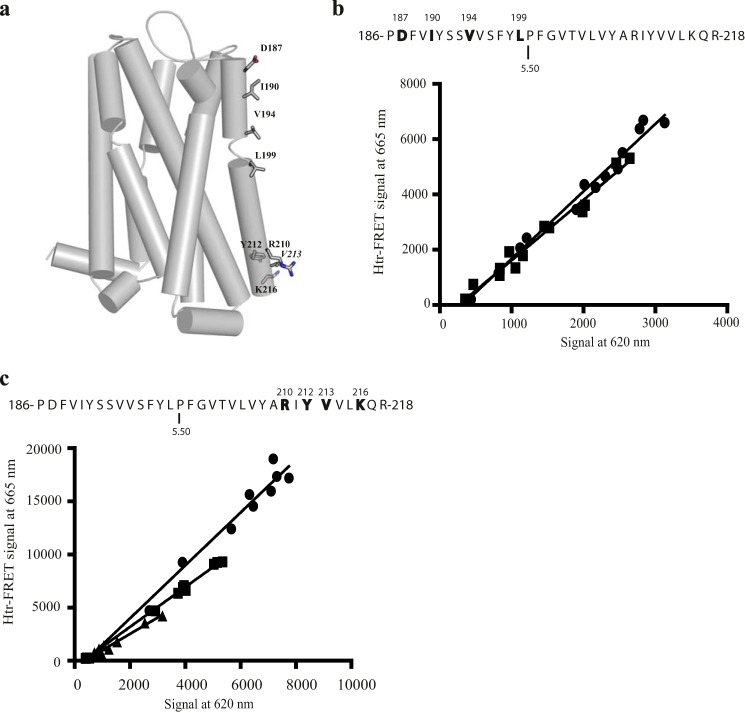
**Role of TMD V in hD_3_-hD_3_ interactions.** *a*, tertiary structure of hD_3_ with TMD V residues that were mutated to alanine shown as sticks. *b* and *c*, the primary structure of TMD V is presented via the one-letter amino acid code. Amino acids that were replaced with alanine are in bold and are denoted by their position in the primary sequence of hD_3_. Proline 5.50 is also indicated. HtrFRET assays performed in HEK293T cells transfected with increasing amounts of VSV-SNAP-hD_3_ (*b* and *c*, *circles*) and either Asp-187,Ile-190,Val-194,Leu-199 VSV-SNAP-hD_3_ (*b*, *squares*) or Arg-210, Tyr-212 VSV-SNAP-hD_3_ (*c, square*), or Arg-210, Tyr-212, Val-213, Lys-216 VSV-SNAP-hD_3_ (*c, triangles*) construct. The plots shown were analyzed by linear regression. See Fig. 10 for quantitative analysis.

As supported by the results above, there is general acceptance that residues from TMD I, TMD II, and helix VIII can provide either one of a number of means to generate a dimer of many class A GPCRs or that these regions provide one interface within a more complex homo-oligomeric structure (18, 25, 26, 33). To assess if further potential interfaces observed in crystal structures of certain class A GPCRs might be relevant to the cell surface organization of the hD_3_, we generated further mutants in VSV-SNAP-hD_3_ (Fig. 3). These were designed to provide potential discrimination between the models shown in Fig. 1, *b–d*. These centered on residues in each of TMDs IV, V, and VI. Generally, for all the mutants studied, a reduction of the expression level of the fully *N*-glycosylated form of the receptor was observed compared with VSV-SNAP-hD_3_ (data not shown). Reduction of cell surface expression of all of these variants was also observed (data not shown). To consider a potential role for TMD V residues, each of Asp-187,Ile-190,Val-194,Leu-199 (residue positions 5.37, 5.40, 5.44, and 5.49) VSV-SNAP-hD_3_, based on contacts reflecting a μ-opioid receptor-like (26) (Fig. 1*d*) or a CXCR4-like (27) (Fig. 1*c*) dimer, or Arg-210,Tyr-212 VSV-SNAP-hD_3_ and Arg-210,Tyr-212,Val-213,Lys-216 VSV-SNAP-hD_3_ (residue positions 5.60, 5.62, 5.63, and 5.66), based on both μ-opioid receptor-like (26) (residues Tyr-212 and Lys-216) and the β_1_-adrenoreceptor-like (25) (mainly Arg-210) (Fig. 1*b*), possible arrangements (Fig. 7*a*) were then assessed in htrFRET studies. Such studies indicated that the slope of the linear regression of the 665-nm/620-nm correlation for the quadruple mutant Asp-187,Ile-190,Val-194,Leu-199 VSV-SNAP-hD_3_ was not significantly different from wild type (Fig. 7*b*). This suggests that this region does not play an important role in hD_3_ receptor organization and, therefore, that organization akin to that observed in the μ-opioid receptor and/or CXCR4 receptor atomic level structures was unlikely. By contrast, a statistically significant effect on cell surface receptor organization was observed with the combination of mutation of Arg-210 and Tyr-212 (0.71 ± 0.01-fold of wild type; mean ± S.E.; Fig. 7*c*). However, although the more extensive mutant Arg-210,Tyr-212,Val-213,Lys-216 VSV-SNAP-hD_3_ was also clearly impaired in oligomeric organization compared with wild type, this mutant did not display further disruption compared with the double Arg-210,Tyr-212 VSV-SNAP-hD_3_ mutant (Fig. 7*c*).

**FIGURE 8.**
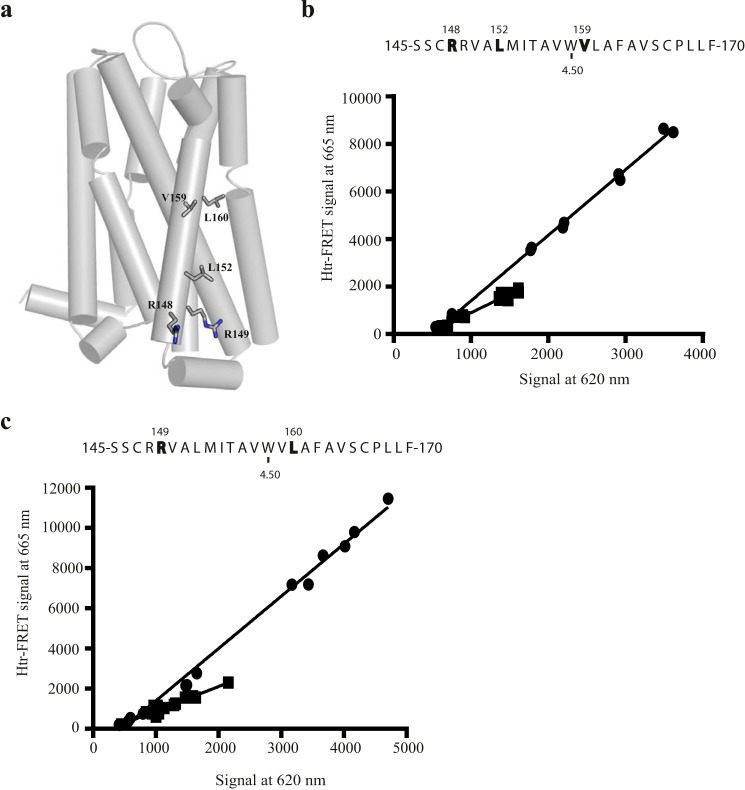
**Role of TMD IV in hD_3_-hD_3_ interactions.** *a*, tertiary structure of hD_3_ with TMD IV residues that were mutated to alanine shown as sticks. *b* and *c*, the primary structure of TMD IV is presented via the one-letter amino acid code. Amino acids that were replaced with alanine are in *bold* and are denoted by their position in the primary sequence of hD_3_. Tryptophan 4.50 is also indicated. HtrFRET assays performed in HEK293T cells transfected with increasing amounts of VSV-SNAP-hD_3_ (*b* and *c*, *circles*) and either Arg-148,Leu-152,Val-159 VSV-SNAP-hD_3_ (*b*, *squares*) or Arg-149,Leu-160 VSV-SNAP-hD_3_ (*c*, *squares*) construct. The plots shown were analyzed by linear regression. See Fig. 10 for quantitative analysis.

**FIGURE 9.**
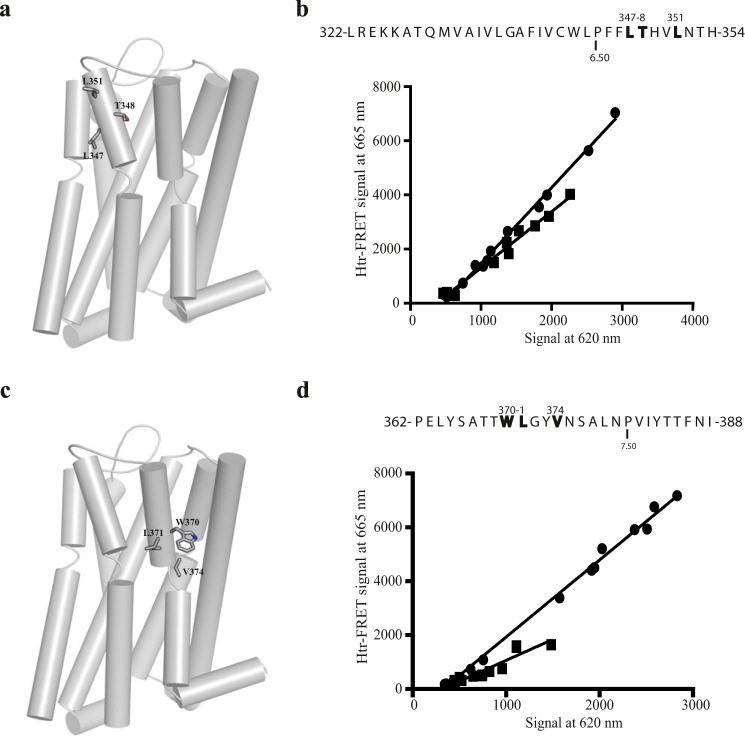
**Roles of TMD VI and TMD VII in hD_3_-hD_3_ interactions.** *a*, tertiary structure of hD_3_ with TMD VI residues that were mutated to alanine shown as sticks. *b*, the primary structure of TMD VI is presented via the one-letter amino acid code. Amino acids that were replaced with alanine are in *bold* and are denoted by their position in the primary sequence of hD_3_. Proline 6.50 is also indicated. *c*, tertiary structure of hD_3_ with TMD VII residues that were mutated to alanine shown as sticks. *d*, the primary structure of TMD VII is presented via the one-letter amino acid code. Amino acids that were replaced with alanine are in *bold* and are denoted by their position in the primary sequence of hD_3_. Proline 7.50 is also indicated. HtrFRET assays performed in HEK293T cells transfected with increasing amounts of VSV-SNAP-hD_3_ (*b* and *d*, *circles*) or Leu-347,Thr-348,Leu-351 VSV-SNAP-hD_3_ (*b*, *squares*), or Trp-370,Leu-371,Val-374 VSV-SNAP-hD_3_ (*d*, *squares*) construct. The plots shown were analyzed by linear regression. See Fig. 10 for quantitative analysis.

In the β_1_-adrenoreceptor crystal dimer TMD V forms part of a dimerization interface that also involves residues from TMD IV (Fig. 1*b*). To assess this model for hD_3_, residues at the equivalent positions in TMD IV predicted from the model, Arg-149 and Leu-160 (residue positions 4.41 and 4.52) or at Arg-148, Leu-152, and Val-159 (residue positions 4.40, 4.44 and 4.51), were mutated (Fig. 8*a*). After transient transfection, htrFRET assays revealed that both Arg-149, Leu-160 VSV-SNAP-hD_3_ and Arg-148,Leu-152,Val-159 VSV-SNAP-hD_3_ had major defects of quaternary structure organization (0.53 ± 0.05- and 0.56 ± 0.03-fold of wild type; mean ± S.E. respectively) (Fig. 8, *b* and *c*). By contrast, in the μ-opioid receptor, TMD V is part of a dimerization interface also involving residues located in TMD VI. Therefore, we also generated the TMD VI mutant Leu-347,Thr-348,Leu-351 (residue positions 6.53, 6.54, and 6.57) VSV-SNAP-hD_3_ (Fig. 9*a*) to mimic residues predicted by this model to be buried upon dimer formation. Interestingly, and in contrast to expectations from the μ-opioid receptor model, based on the lack of effect of mutation (Asp-187,Ile-190,Val-194,Leu-199 VSV-SNAP-hD_3_) of the extracellular side of TMD V (Fig. 7*b*), htrFRET assays here revealed that Leu-347,Thr-348,Leu-351 VSV-SNAP-hD_3_ caused a statistically significant decrease (0.79 ± 0.02-fold; mean ± S.E.) in the signal at 665 nm compared with the wild type receptor at equivalent cell surface expression levels (Fig. 9*b*).

As the htrFRET signal reduction observed for the TMD VI mutant Leu-347,Thr-348,Leu-351 VSV-SNAP-hD_3_ and the lack of effect of the TMD V mutant Asp-187,Ile-190,Val-194,Leu-199 VSV-SNAP-hD_3_ appeared to exclude either a possible μ-opioid receptor-like dimer arrangement (Fig. 1*d*) or a CXCR4-like arrangement (Fig. 1*c*), we considered other possible hD_3_ oligomer arrangements including those that predict tetrameric organization. Recently, mathematical analysis of spectrally resolved multi-photon FRET microscopy data has provided evidence that a substantial proportion of the human M_3_ muscarinic acetylcholine receptor is present at the surface of transfected cells as a tetramer with rhombic organization (39). Moreover, as in-house data had shown a specific role for both TMD VII and TMD VI in organization of the tetramer via cholesterol molecules that bridge a pair (dimer + dimer) of TMD I-helix VIII interface M_3_ muscarinic receptor dimers (40), we generated both additional mutants and models of potential organization of the hD_3_, akin to these muscarinic M_3_ models, to explore if these could unify the experimental observations.

**FIGURE 10.**
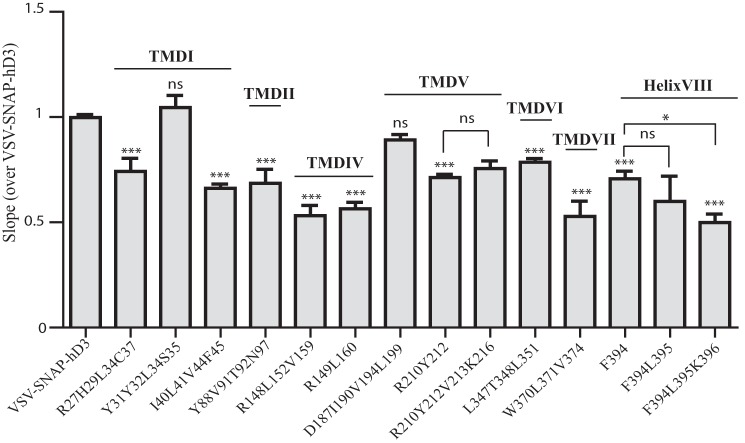
**Many regions of the helical domains of hD_3_ contribute to effective oligomerization.** The slope values of 665 nm over 620 nm fluorescence emission for each mutant described in Figs. 5–9 were normalized to those obtained with VSV-SNAP-hD_3_ (which was included as control in each individual experiment). Data are the means ± S.E. of at least three independent experiments. Statistical analysis was performed by one-way analysis of variance, with Dunnett's test for multiple comparisons where appropriate, for example when comparing VSV-SNAP-hD_3_, Phe-394,Leu-395 VSV-SNAP-hD_3_, and Phe-394,Leu-395,Lys-396 VSV-SNAP-hD_3_ to Phe-394 VSV-SNAP-hD_3_. *ns*, not significant. *p* < 0.05 (*) and *p* < 0.0001 (***) compared with VSV-SNAP-hD_3_ or to the indicated receptor. Mutants that produce a significant reduction in the slope are predicted to contain residues that contribute to the organizational structure of hD_3_.

Based on these models a TMD VII mutant Trp-370,Leu-371,Val-374 (residue positions 7.40, 7.41, and 7.44), VSV-SNAP-hD_3_ was produced (Fig. 3) and studied (Fig. 9, *c* and *d*). Both total (not shown) and cell surface expression level of Trp-370,Leu-371,Val-374 VSV-SNAP-hD_3_ were reduced compared with wild type. Although expressed poorly at the cell surface, htrFRET studies again generated a linear regression for oligomerization *versus* cell surface expression. Most importantly, the slope of the linear regression for Trp-370,Leu-371,Val-374 VSV-SNAP-hD_3_ was reduced markedly (0.53 ± 0.07-fold; mean ± S.E.) compared with the wild type hD_3_ receptor (Fig. 9*d*). These results, which are not predicted by any of the crystal structure dimer models are, however, fully consistent with the rhombic tetramer model. Mapping of the full htrFRET experimental data set (Fig. 10) back to such a model also resulted in predictions of effects of mutants as observed from the experimental studies (see “Discussion”).

## Discussion

There are five distinct, but highly related (D_1–5_), receptors that respond selectivity to the catecholamine dopamine. These have been reported to form both homomers and heteromers with partners within this subfamily (12, 13, 15, 36, 41) and also with GPCRs, which respond to different ligands, for example, receptors for adenosine (42). At the level of homomeric interactions there has been particular focus on members of the D_2_-like subfamily (D_2_, D_3_, and D_4_). The capacity of the dopamine D_2_ receptor to form homodimers and higher order oligomers in living cells has been studied extensively by Javitch and co-workers (16–18). A symmetric interface of hD_2_ receptor dimerization was described as involving TMD IV in the active state of the receptor and both TMD IV and TMD V in the inactive (16, 17). However, the quaternary structure for hD_2_ receptor has also been suggested to be composed of at least four protomers in which interactions occur both between residues from TMD IV and TMD V and by residues at the extracellular site of TMD I and residues from helix VIII (18). Although the high relatedness of D_2_ and D_3_ receptors might suggest similar means of generating homomeric interactions, this has not been assessed directly, and much less is known about the basis of D_3_ receptor homo interactions. Therefore, in the current studies the use of computational models and experimental studies were combined to investigate these questions. Residues predicted as possibly being involved at oligomer interfaces were assessed by use of alanine mutagenesis, and their effect was measured by use of htrFRET assays employing Tag-Lite^TM^ technology (21). As the Tag-Lite^TM^ htrFRET energy acceptor and donor moieties are not cell-permeant and link covalently to the SNAP tag that was introduced into the extracellular N-terminal domain of wild type VSV-SNAP-hD_3_ and the various receptor mutants studied, signals reflected only cell surface-delivered receptors. Indeed, measuring fluorescence emission of SNAP-Lumi4-Tb at 620 nm after excitation at 337 nm provided a direct measure of cell surface expression without concern that the mutations might affect the affinity of radioligands in direct binding studies. Despite this, we also assessed the possible effects of the mutations on the affinity of the constructs to bind the antagonist [^3^H]spiperone. Only a single TMD I mutant showed a large change in affinity for this ligand. The one mutant we describe that lost affinity for [^3^H]spiperone to the extent that we were no longer able to measure this effectively was Ile-40,Leu-41,Val-44,Phe-45 (1.43, 1.44, 1.47, 1.48) VSV-SNAP-hD_3_, one of the most extensive mutants we generated. Here four predominantly hydrophobic residues were each altered to alanine. These are located in the middle of TMD I. Based on a variety of atomic level structures, TMD I is not routinely an element that makes specific interactions with small molecule ligands, and indeed, in the available atomic level structure of the D_3_ receptor eticlopride does not interact directly with these residues (20). As such, a potential explanation for the loss of affinity of [^3^H]spiperone for this mutant must be speculative. However, as defined in the models, Leu-1.44 interacts both with the same residue of the other protomer and with Ile-1.43, Val-1.47, and Phe-1.48 via a cholesterol molecule. Disruption of the contribution of cholesterol, a key overall element of the models we generated, may be vital to the effect on ligand binding. It is worth noting, however, that although we were unable to directly measure the affinity of [^3^H]spiperone for this mutant, clearly it does still bind this ligand with significant affinity because treatment of cells with non-radiolabeled spiperone promotes more effective cell surface delivery of the expressed mutant,^6^ a feature generally referred to as a “pharmacological chaperone” effect. This requires the receptor variant to be able to bind the ligand in question. The other mutants, with the possible exception of Arg-148,Leu-152,Val-159 VSV-SNAP-hD_3_, displayed only modest effects, indeed no more than 4-fold, on the measured binding affinity of [^3^H]spiperone. Even these measured differences may represent something of an artifact. Measures of binding affinity can be modified if expression levels of receptor variants are markedly different, and the results of Fig. 4 show that this was clearly the case for a number of the mutants studied.

The SNAP-tag approach proved to be vital for analysis as virtually all of the receptor mutants studied were delivered to the cell surface of transfected cells less well than the wild type receptor construct. However, quantification of the extent of cell surface delivery allowed measurement of energy transfer and, therefore, comparison of protein-protein interaction effectiveness, at equal levels of cell surface expression.

Despite many of the hD_3_ variants displaying significant alteration in protein-protein interactions, for none of these was homo-oligomerization completely ablated. We considered a number of scenarios that could account for this. First, as variants that were more extensive than quadruple point mutants were simply not expressed at the cell surface, it was possible that we had only targeted part of more extended interacting dimer surfaces. Second, as experimental data and crystal structures suggested the potential for multiple dimer interfaces, mutations in a single TMD might disrupt only a subset of the existing dimers. However, because we showed directly that at least two different interfaces of dimerization exist for the hD_3_ and because both we and others have shown that class A GPCRs can form higher order oligomers (18, 38, 43–45), we also considered if such models could provide a single, coherent explanation for the overall data set.

**FIGURE 11.**
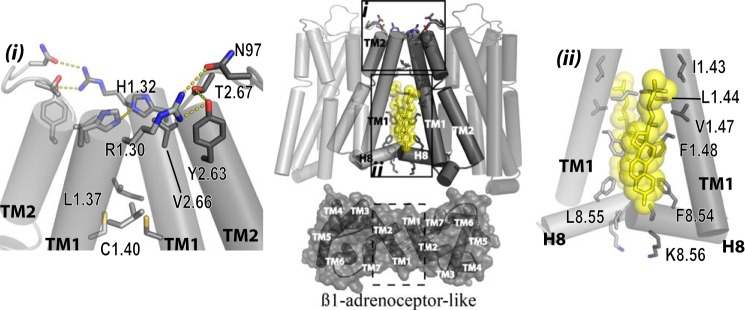
**Molecular modeling of potential dimeric arrangement: the TMD I-TMD II-helix VIII interface.** *Center panel*, general view of a model of an hD3 dimer generated by TMD I-TMD II and helix VIII residues (*opaque light* and *dark gray*) as the interacting interfaces (upper panel) and organized with a β_1_-adrenoreceptor-like arrangement (*lower panel*). Residues in *gray sticks* are those that produced htrFRET reduction when mutated to alanine. *Yellow sticks* and *spheres* show cholesterols as observed in β_2_-adrenoreceptor and serotonin 5-HT_2B_ atomic level receptor structures. *Inset (i)* indicates residues from the extracellular side of TMD I and TMD II as well as external loop 1 that when mutated reduce htrFRET. Arg-1.30 from 1 hD_3_ protomer forms hydrogen bonds with Tyr-2.63, Thr-2.67, and Asn-97 of external loop 1. His-1.32 interacts with the same residue of the other protomer. Leu-1.37 and Cys-1.40 form a hydrophobic interaction between protomers. *Inset (ii)* shows details of the intracellular side of TMD I and helix VIII. Leu-1.44 interacts both with the same residue of the other protomer and with Ile-1.43, Val-1.47, and Phe-1.48 via a cholesterol molecule (*yellow sticks* and *sphere*). Phe-8.54, Leu-8.55, and Lys-8.56 form an extended interacting surface between helix VIII from each monomer.

Although crystal structures of class A GPCRs show different interfaces to be involved in dimer organization, a rather conserved interface, involving TMD I, TMD II, and intracellular helix VIII is a routine feature (25–26, 33–34). A model of a hD_3_ homodimer based on this arrangement is shown in Fig. 11, *central panel*, and compared with the observed structure of the β_1_-adrenoreceptor. Generating the Arg-27^1.30^,His-29^1.32^,Leu-34^1.37^,Cys-37^1.40^ mutant in the extracellular side of TMD I and Tyr-88^2.63^,Val-91^2.66^,Thr-92^2.67^,Asn-97^w+1^ at the top TMD II (Asn-97 is part of extracellular loop 1 rather than within the TMD), which were predicted to form a hydrogen-bond network that stabilizes hD_3_-hD_3_ interactions (Fig. 11, *inset (i)*) showed that both sets of alterations had a substantial impact on the quaternary structure of the receptor. Of note, residues at similar positions in both TMD I, namely, Gln-1.29 (position 1.30 is an alanine), Glu-1.32, Leu-1.37, and Ala 1.40 as well as in TMD II Thr-2.63, Val-2.66, and Arg-2.67 and a leucine one residue after the strongly conserved tryptophan (W+1) (46) in external loop 1, have been shown to form a packed interaction in the β_1_-adrenoreceptor (25). As studies on hD_2_ receptor dimerization have indicated that residues at positions 1.34, 1.35, and 1.38 have an active role (18), we also investigated the effect of mutating the sequence Tyr-31^1.34^,Tyr-32^1.35^, Leu-34^1.37^,Ser-35^1.38^ on hD_3_ interactions. However, this variant was both well expressed at the cell surface and showed no disruption of quaternary organization. Indeed, our model does not predict a role for these residues in the hD_3_ receptor (Fig. 11) as these either face TMD VII of the same protomer (Tyr-32^1.35^) or face the external side close to it (Tyr-31^1.34^ and Ser-35^1.38^). Further work on the D_2_ receptor will be needed to understand these potential discrepancies. Our models also predicted possible roles of residues in the lower half and toward the cytoplasmic end of TMD I. A strong effect on both the surface expression level and on quaternary structure of hD_3_ receptor was indeed observed for the mutant Ile-40^1.43^, Leu-41^1.44^, Val-44^1.47^, Phe-45^1.48^ hD_3_. Although an important role of leucine 1.44 can be explained via a symmetrical hydrophobic interaction with the same residue in the other protomer (Fig. 11, *inset (ii)*), as was also observed in the β_1_-adrenoreceptor dimeric crystal (25), contribution of the residues at position 1.43 (Ile-40), 1.47 (Val-44) and 1.48 (Phe-45) cannot be explained via direct interaction between the two protomers. However, the corresponding residues in these positions are involved in binding a molecule of cholesterol in the crystal structures of both the β_2_-adrenoreceptor (34) and the serotonin 5-HT_2B_ receptor (47), and a molecule of cholesterol in this location in the hD_3_ would provide a bridge between the helices of the two protomers (Fig. 11, *inset (ii)*). Moreover, the contribution of cholesterol and/or other lipid molecules to GPCR organizational structure may be widespread, as further structures of class A GPCRs contain other, sometimes structurally conserved, molecules of cholesterol. Moreover, in an experimental paradigm Oates *et al.* (48) have shown cholesterol to influence activity, stability, and oligomerization of the neurotensin NTS1 receptor. Finally, the dimer of the seven TMD region of the class C metabotropic glutamate receptor 1 (49) shows cholesterol molecules making a specific contribution to the receptor-receptor interface. Such data and observations resulted in us explicitly considering the possible importance of cholesterol molecules in our models by adding them to hD_3_ monomeric units (see “Materials and Methods”). Of course these suggestions of specific roles for molecules of cholesterol are inherently speculative, not the least because means to deplete specific molecules of cholesterol, rather than the bulk cholesterol population, are lacking. Despite this, the implications of the identified positions of molecules of cholesterol in GPCR structures is intriguing and worthy of further investigation.

As noted earlier, a number of studies have implied roles for elements of the intracellular helix VIII in GPCR dimer interactions. Alanine mutation of Phe-394^8.54^ in hD_3_ receptor produced a substantial effect on quaternary structure without altering cell surface expression of the receptor. This effect was further increased with the simultaneous mutation of Leu-395^8.55^ and Lys-396^8.56^. Residues at position 8.54, 8.55, and 8.56 in both β_1_-adrenoreceptor and in μ-opioid receptor structures have also been observed to be part of the extended TMD I-TMD II-helix VIII interface (25, 26).

**FIGURE 12.**
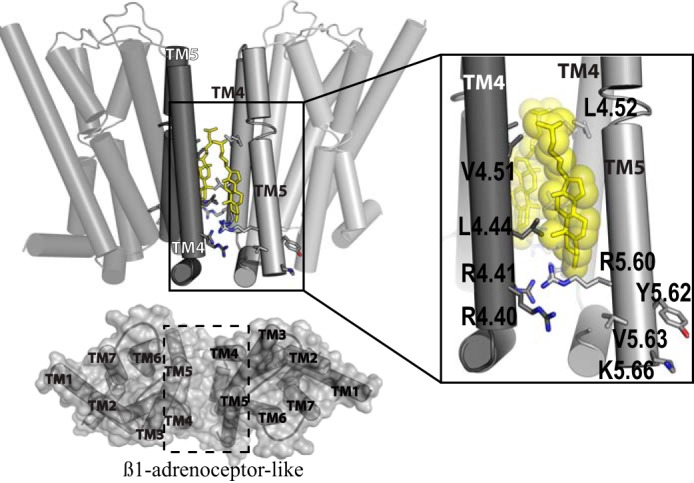
**Molecular modeling of potential dimeric arrangement: the TMD IV-TMD V interface.** *Left panel*, general view of a model of a hD_3_ dimer that employs residues from TMD IV and TMD V (*opaque light* and *dark gray*) as interacting surfaces (*upper panel*) and organized with a β_1_-adrenoreceptor-like arrangement (*lower panel*). Residues in *gray sticks* when mutated to alanine induce htrFRET reduction; *yellow sticks* indicate a possible cholesterol molecule mediating the interaction between protomers. *Right panel*, detail of the interaction between protomers. Residues of TMD IV extensively interact with a cholesterol molecule, positioned as observed in the P2Y_12_ receptor atomic level structure, whereas only Arg-5.60 is actively involved in this interacting interface (indeed adding Val-5.63 and Lys-5.66 to the Arg-5.60 and Tyr-5.62 mutant did not further reduce htrFRET).

In contrast to the rather conserved interface involving TMD I-TMD II, and helix VIII, less conserved interfaces are observed in crystal structures on the “opposite” side of the receptor TMD bundle, although each of these points to a pivotal role of TMD V. hD_3_ receptor variants in this helix were engineered that simultaneously substituted those residues predicted to be selectively involved only in one of the TMD V-based dimers observed in the atomic level structures. A hD_3_ dimer with CXCR4-like organization (27) predicted TMD V residues Asp-187^5.37^,Ile-190^5.40^,Val-194^5.44^,Leu-199^5.49^ to be crucial for the interface. However, the htrFRET signal per copy of cell surface receptor for this mutant was not different from wild type, thus excluding this type of organization. In a similar way, a hD_3_ dimer organized with a μ-opioid receptor-like TMD V-TMD VI configuration (26) was also excluded because the crystal structure and models indicated a role for Asp-187^5.37^ (already noted from the CXCR4-like TMD V mutant not to be an important contributor) as well as from Tyr-212^5.62^ and Lys-216^5.66^. The addition of alanine substitutions of both Leu-213^5.63^ and Lys-216^5.66^ into a mutant (Arg-210^5.60^ and Tyr-212^5.62^ hD_3_) that had a strong effect to reduce the htrFRET signal did not result in further reduction of the htrFRET signal. By contrast, the mutagenesis studies were most consistent with an hD_3_ dimer organization akin to that observed for the β_1_-adrenoreceptor (25). Such a β_1_-adrenoreceptor-like configuration indicated roles of residues from both TMD V (in particular residue Arg-210^5.60^) and TMD IV. Indeed, mutation of residues Arg-148^4.40^, Arg-149^4.41^, Leu-152^4.44^, and Val-159^4.51^, Leu-160^4.52^ in TMD IV had large effects on hD_3_ receptor quaternary organization. Involvement of TMD IV and TMD V in GPCR quaternary structure has also been predicted from biochemical studies. For example, when investigating the basis of hD_2_ homodimer interactions using a chemical cross-linking approach, residues from TMD IV (including Arg-4.41, Val-4.44, Val-4.51, Leu-4.52, which are equivalent to Arg-149, Leu-152, Val-159, and Leu-160 in hD_3_) and TMD V were described as important in maintaining the stability of the hD_2_-hD_2_ interaction (17). Similarly for the δ-opioid (50) and 5-HT_1A_ (51) receptors, residues at positions 4.40 and 4.41 (equivalent to Arg-148 and Arg-149 in hD_3_) were shown to be part of a suggested TMD IV-TMD V interface. Furthermore, the P2Y_12_ purinoceptor (35) may also form a TMD V dimer mediated by cholesterol molecules. Indeed, the predicted hD_3_ model, based on β_1_-adrenoreceptor TMD IV-TMD V dimer organization, is consistent with a cholesterol-mediated dimer. Mutants that reduced the htrFRET signal, including residues Arg-148^4.40^, Leu-152^4.44^, and Val-159^4.51^ in TMD IV as well as Arg-210^5.60^ from TMD V, are compatible with an interaction involving a cholesterol molecule in the equivalent position as found in the P2Y_12_ purinoceptor structure (Fig. 12).

These results thus can account for at least two different dimeric arrangements of hD_3_ receptor, both in broad agreement with those observed in the β_1_-adrenoreceptor crystal structure (25). Based on such dimer interfaces, we constructed models of possible tetrameric organization resulting from dimer + dimer contacts and examined predictions that might discriminate between these by considering both rhombic and “linear” tetramer models. Both TMD I-TMD II-helix VIII and TMD IV-TMD V interfaces can be simultaneously involved in a linear dimer + dimer (as hypothesized for the β_1_-adrenoreceptor (25) and for the μ-opioid receptor (26)), and importantly, residues from other TMDs are not required to allow such organization. By contrast, this is not the case in rhombic dimer + dimer organization, which also requires contributions of other helices. We thus built possible rhombic dimer + dimer tetramers based on shape complimentarily of the monomer of the antagonist-bound inactive D_3_ receptor structure. The models that incorporated pairs of dimers in which each dimer interface was between TMD IV and TMD V resulted in forms that were unable to simultaneously bind two heterotrimeric G proteins in their nucleotide “empty” configuration (see “Materials and Methods”).

**FIGURE 13.**
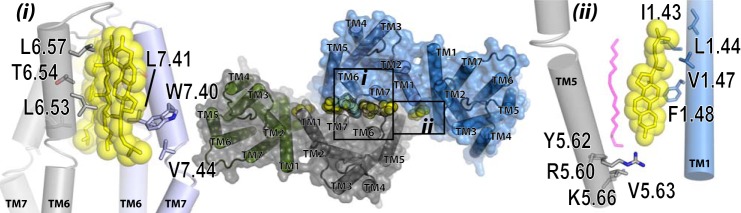
**Molecular modeling of hD_3_ in tetrameric arrangements.** Model of hD_3_ in a tetrameric arrangement as result of a dimer + dimer interactions. Each dimer is shown as a semi-transparent surface, whereas predicted cholesterols are shown as *yellow spheres* forming a buffer between the two dimers. *Inset (i)* shows details of the TMD VI and TMD VII interface, and the residues shown in *sticks* (*gray* and *light blue*) were found experimentally to affect hD_3_ quaternary structure. *Yellow sticks* and *spheres* depict predicted cholesterol molecules in positions as observed in adenosine A_2A_ receptor, μ-opioid receptor, and the P_2_Y_12_ receptor structures. *Inset (ii)* shows details of the predicted interaction between TMD V Arg-5.60 and Tyr-5.63 (in *gray sticks*) of one dimer and the TMD I cholesterol (in *yellow sticks* and *spheres*). A predicted palmitoyl moiety, bound to Cys-8.60, is also shown in *magenta semi-transparent sticks*.

When employing TMD I-TMD II-helix VIII interface dimers a tightly packed rhombic tetramer was produced (Fig. 13, *central panel*). This complex could simultaneously bind two heterotrimeric G proteins in their nucleotide-free form. Significantly this model (Fig. 13, *inset (ii)*) predicted an important role for TMD V but, rather than at the protomer-protomer interface of the individual dimers, its role was at the dimer + dimer interface of the rhombic tetramer. This model shows cholesterol binding to the lower part of TMD I and mediating an interaction of TMD I from one dimer with TMD V from the second dimer that specifically involves residues Arg-210^5.60^ and Tyr-212^5.62^ (Fig. 13, *inset (ii)*). Mutation of these residues disrupted quaternary organization at the cell surface. Perhaps even more significantly, the most extensive predicted “dimer + dimer” interface in the rhombic tetramer model involved residues from TMD VI and from TMD VII (Fig. 13). No role of TMD VI and TMD VII in hD_3_ quaternary structure is predicted in the linear tetramer model involving the two dimer interfaces we found experimentally. These predictions allowed direct experimental comparison of linear *versus* rhombic tetramer models, as they predicted markedly different outcomes for mutants in TMD VI and TMD VII on htrFRET signal and quaternary structure. The TMD VI mutant Leu-347^6.53^,Thr-348^6.54^,Leu-351^6.57^ and, particularly, the TMD VII mutant Trp-370^7.40^,Leu-371^7.41^,Val-374^7.44^ displayed marked reduction in hrtFRET signal, consistent with these alterations affecting quaternary structure and, therefore, providing support for the rhombic tetramer model.

Interestingly, two molecules of cholesterol that interact with TMD VI at its extracellular side in the rhombic tetramer constructs create a layer of four cholesterol molecules that line up to form a “buffer” between the dimers (Fig. 13). Of note, a cholesterol located at the extracellular side of TMD VI in an adenosine A_2A_ receptor crystal (52) is superimposed, after building the rhombic tetramer hD_3_ model, with the cholesterol observed on the extracellular side of TMD VII of the P2Y_12_ receptor (36). A possible direct TMD VI-TMD VII dimer was also dismissed from further consideration both because such hypothetical dimers would impede the well known outward displacement of TMD VI upon ligand-induced activation and subsequent heterotrimeric G protein coupling and because such an interface has not been observed in any crystal structure to date. Interestingly, Leu-371^7.41^ and Val-374^7.44^ lie deep in the concave spot of helix TMD VII, a location from which they would be unlikely to form direct residue-residue interactions with TMD VI. However, the model predicts they can do so via the tail of an intermediate molecule of cholesterol (Fig. 13, *inset (i)*).

Taken together, these results suggest not only the capability of hD_3_ to form dimers but also higher order oligomers in which four protomers are predicted to organize in a rhombic arrangement. It is notable, therefore, that mathematical analysis of FRET efficiency peaks taken from spectrally resolved, multi-photon imaging of cells expressing a pair of FRET-competent forms of the M_3_ muscarinic acetylcholine receptor has also predicted that a substantial proportion of the receptor is organized within such rhombic tetramers (39). It also suggests why mutations in a single TMD are unable to result in elimination of htrFRET signal.

Whether this is the basic default position of class A GPCRs in general remains to be established, as does the stability of such tetramers and their importance for allosteric ligand effects and, potentially, for ligand signaling and bias. If these are not stable complexes, and certain studies have suggested that GPCR “dimers” may rapidly associate and then dissociate (53, 54), then information generated in these studies may be utilized to develop peptides able to selectively disrupt dimers, as in studies on the secretin receptor (55), or tetramers, providing the possibility to assess their functional relevance in living cells.

## Footnotes

6 S. Marsango and G. Milligan, unpublished information.

## Footnotes

5 The abbreviations used are:

GPCR: G protein-coupled receptor
hD_3_: human dopamine D_3_
htrFRET: homogeneous time-resolved FRET
TMD: transmembrane domain
EGFR: epidermal growth factor receptor
VSV: vesicular stomatitis virus.
